# Comparison of Intercostal and Subxiphoid Left Pleural Drain After Coronary Artery Bypass Grafting (CABG) Surgery: A Systematic Review and Meta-Analysis

**DOI:** 10.7759/cureus.61710

**Published:** 2024-06-05

**Authors:** Pranay Mehsare, Nitinkumar Borkar, Nitin Kumar Kashyap, Gaind Saurabh, Nirupam Chakraborty

**Affiliations:** 1 Cardiothoracic Surgery, All India Institute of Medical Sciences, Raipur, Raipur, IND; 2 Pediatric Surgery, All India Institute of Medical Sciences, Raipur, Raipur, IND

**Keywords:** intercostal drain, subxiphoid drain, pleural drainage, arterial grafts, cabg

## Abstract

Coronary artery bypass grafting (CABG), a prevalent surgery for coronary artery disease, often involves left internal mammary artery harvesting, necessitating left pleural drain insertion. This can lead to pain, discomfort, and respiratory issues. This analysis compares outcomes between subxiphoid and intercostal left pleural drain insertion. Following the Preferred Reporting Items for Systematic reviews and Meta-Analyses (PRISMA) guidelines, this systematic review and meta-analysis involved searches in MEDLINE, Cochrane Central Register of Controlled Trials (CENTRAL), Scopus, Google Scholar, and Clinical Trial Registry. Studies were selected based on primary outcomes (postoperative ventilator support duration and pain score) and secondary outcomes (percentage predicted vital capacity (VC), forced VC (FVC), and partial pressure of oxygen (PO2) in arterial blood gas (ABG) analysis). Statistical analysis used a random effect model, pooled risk ratio, and I2 heterogeneity. Nine studies (seven randomized and two nonrandomized) with 412 patients met the inclusion criteria. Pooled analysis indicated reduced ventilation time and postoperative pain with the subxiphoid drain compared to the intercostal drain. Spirometry parameters showed improved VC, FVC, and PO2 in ABG analysis. This meta-analysis suggests that subxiphoid pleural drain insertion in CABG patients is associated with shorter ventilation times, lower pain scores, and improved pulmonary function compared to intercostal drain placement.

## Introduction and background

Coronary artery bypass grafting (CABG) surgery stands as the most frequently performed procedure by cardiac surgeons today [1]. A wide range of vascular conduits are available for CABG, with the primary options being the internal thoracic artery (ITA), saphenous vein, and radial artery [2].

After nearly four decades of coronary artery bypass surgery, ITA grafts have emerged as the superior conduit for revascularization. They demonstrate resistance to arteriosclerosis and offer exceptional long-term patency. Notably, employing the left ITA to revascularize the left anterior descending artery yields improved long-term survival and reduced cardiac events [3].

However, in the majority of cases, harvesting the left ITA pedicle necessitates opening the left pleural cavity, resulting in the need for drainage. CABG itself is associated with impaired pulmonary function in the postoperative phase, contributing to increased postoperative morbidity [4]. The pleurotomy required for this procedure, coupled with the presence of a pleural drain, adds to patient discomfort and negatively impacts respiratory mechanics [5,6].

These drains serve several purposes, including minimizing fluid accumulation in the pleural cavity, monitoring bleeding, and preventing potential complications such as pericardial effusion, hemothorax, and tamponade [7]. They are inserted either in the subxiphoid region or the intercostal space. The subxiphoid drain is directed toward the base of the left lung, while the intercostal drain is placed in the fifth or sixth intercostal space and directed toward the apex of the lung [8].

While essential, the presence of these drains can lead to alterations in pulmonary function, restricting the activity of respiratory muscles, modifying ventilatory mechanics, and causing intense pain and postoperative discomfort [8,9]. Several factors, such as general anesthesia, sternotomy, cardiopulmonary bypass, ITA grafts with pleurotomy, and pleural drain placement, can influence pulmonary function and clinical outcomes in CABG patients [10].

Recent evidence suggests that inserting the pleural drain in the subxiphoid position, rather than the intercostal space, minimizes chest wall trauma and preserves respiratory function during the early postoperative period [11,12]. Studies by Guden et al. [13] and Simon et al. [14] reported that clinical outcomes remain similar in both groups of patients. Hagl et al. [12], Ozelami Vieira et al. [15], Cancio et al. [16], Guizilini et al. [11,17] and Elnasr et al. [8] have reported positive outcomes associated with subxiphoid drain insertion, including reduced impairment of lung function, lower subjective pain, and improved recovery parameters.

This study systematically compares the outcomes of subxiphoid and intercostal pleural drain placements after CABG surgery.

## Review

Materials and methods

This systematic review and meta-analysis followed the Preferred Reporting Items for Systematic reviews and Meta-Analyses (PRISMA) guidelines [18]. We initially conducted a literature search on PubMed and Google Scholar to ensure the absence of similar published meta-analyses.

A comprehensive and systematic search was then performed in electronic databases, including MEDLINE, Cochrane Central Register of Controlled Trials (CENTRAL), Scopus, and Google Scholar. The authors conducted this search independently and extended it up to December 31, 2023. Additionally, we searched the Clinical Trial Registry (ClinicalTrials.gov) and major conference proceedings up to the same date without imposing any language restrictions. We reran the searches before the final analysis to capture any updated or newly available data.

The search terms employed were as follows: (intercostal drain OR pleural drain OR subxiphoid drain OR drain) AND (coronary artery bypass surgery (CABG)). We also conducted manual searches by reviewing related references provided in the identified studies.

Eligibility criteria

In our meta-analysis, we considered randomized controlled trials and comparative studies. The study participants consisted of patients who had undergone CABG surgery. We included studies where intercostal left pleural drain insertion was the surgical intervention and the comparator was subxiphoid left pleural drain insertion. Patients who had undergone emergency CABG and surgery without left pleural drain insertion were excluded from our analysis.

The primary outcome of our study was the time required for ventilator support postoperatively and postoperative pain scores. The secondary outcome was postoperative spirometry parameters, including partial pressure of oxygen (PO2) in arterial blood gas (ABG), a decrease in percentage predicted vital capacity (VC), and a decrease in percentage predicted forced VC (FVC).

These criteria were used to ensure that the selected studies were relevant to our research objectives and allowed us to assess the impact of intercostal and subxiphoid left pleural drain insertion on the specified outcomes.

Data collection and analysis

Study Selection

**Figure 1:**
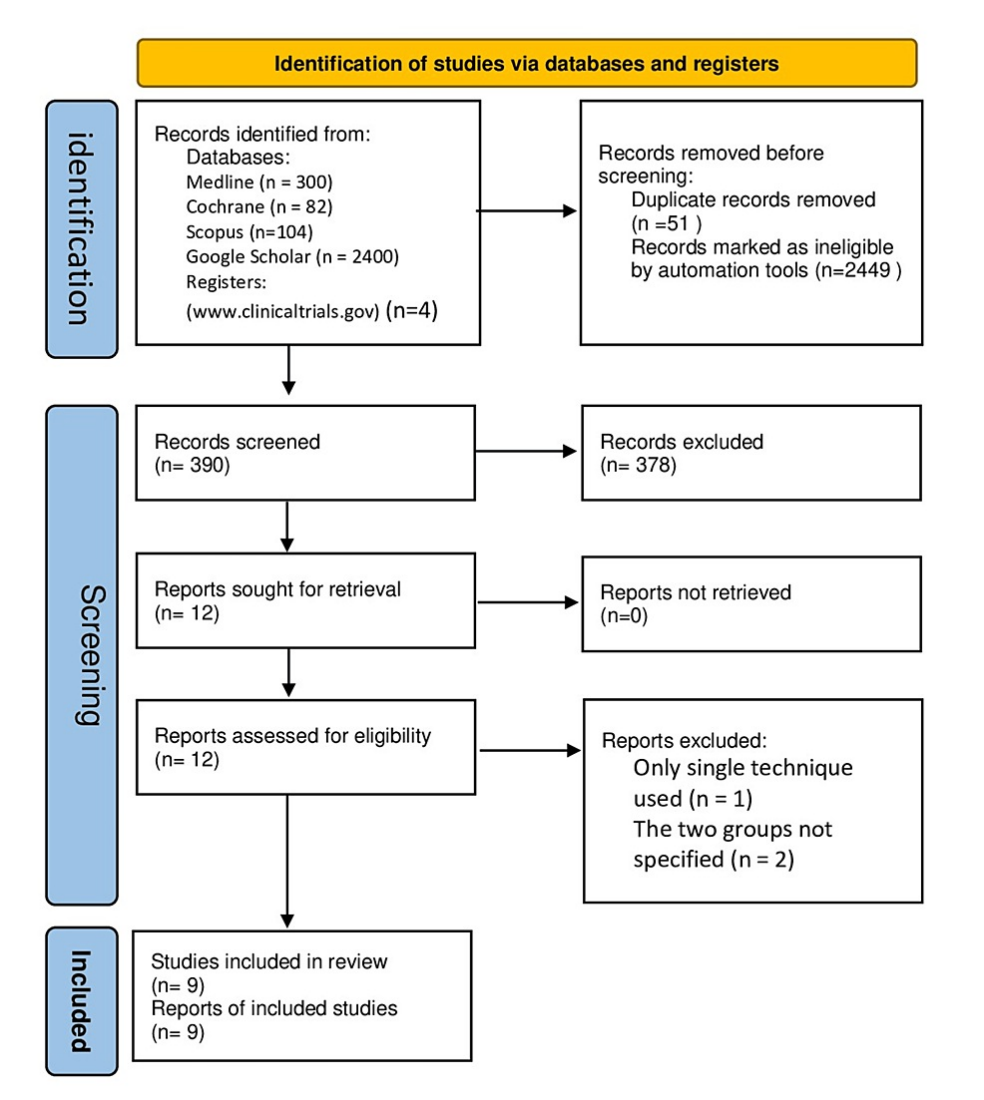
PRISMA flow diagram PRISMA, Preferred Reporting Items for Systematic reviews and Meta-Analyses

The authors conducted an independent review of the abstracts and titles of the identified articles. All potentially relevant articles were thoroughly assessed by reviewing the full text. After eliminating any duplicate publications from the search results, the full texts of potentially eligible studies were retrieved. The authors independently evaluated these full-text articles to determine their eligibility for inclusion (Figure 1).

Methodological Quality Assessment and Risk of Bias Assessment of Included Studies

To assess methodological quality, the authors employed the Modified Downs and Black scale [19]. Additionally, we calculated the Kappa coefficient to estimate the inter-rater reliability for the included studies using the Modified Downs and Black scale [20]. The authors independently assessed bias using the Cochrane Collaboration tool, specifically designed for randomized studies [21].

Data Extraction

Upon selecting the relevant studies, data extraction was carried out independently by the authors. The extracted data included baseline information for each study, such as author information and year of publication, as well as the number of patients in each study, the number of patients in each group, the mean or median age, and the age range of the included patients. Furthermore, the specified outcome measures were also extracted and recorded in a data extraction table using MS Excel (version 16.16.27; Microsoft Corporation, Redmond, Washington, United States). Any discrepancies between the two observers were resolved through consensus and consultation with the senior author.

Statistical Analysis

Continuous outcome variables were presented as mean differences (MDs) with corresponding 95% CIs. The individual participant was considered the preferred unit of analysis. To evaluate the heterogeneity among the studies, a visual assessment of the CI in the forest plot (eyeball test) was performed. Additionally, we quantified heterogeneity using the I2 statistics. The interpretation of I2 values followed these ranges: 0-40%: may not be significant; 30-60%: may indicate moderate heterogeneity; 50-90%: may suggest substantial heterogeneity; and 75-100%: indicates considerable heterogeneity [22].

These analyses were conducted to assess the variation among the included studies and to guide the interpretation of heterogeneity levels in the meta-analysis.

Results

The initial literature search yielded a total of 2,890 studies, with contributions from different sources: 300 from PubMed, 82 from Cochrane, 104 from Scopus, 2,400 from Google Scholar, and four from ClinicalTrials.gov. After removing duplicate studies, a total of 390 records were screened based on their titles and abstracts. Subsequently, 12 studies were selected for a full-text review, and from these, nine studies were included in the evaluation (comprising seven randomized and two nonrandomized studies), as illustrated in Figure 1.

Study Characteristics

A summary detailing the characteristics of the included studies can be found in Table 1.

**Table 1:** Summary of baseline characteristics of all studies RCT, randomized controlled trial

Serial number	Studies	Setting	Study period	Design	Number of patients (N)	Mean age (years)	Pulmonary function assessment	Outcome
1	Hagl et al. [12]	Hannover Medical School, Hannover, Germany	1999	RCT	Total (N) = 30	NA	First, third, and fifth day	The subxiphoid drain better preserves lung function compared to the intercostal drain.
					Group 1 (subxiphoid) = 15	57.8 + 11.2 years		
					Group 2 (intercostal) = 15	60.7 + 9.4 years		
2	Ozelami Vieira et al. [15]	Hospital de Clínicas da Faculdade de Medicina da Universidade do Triângulo Mineiro, Uberaba, Minas Gerais, Brazil	January 2010 to July 2011	RCT	Total (N) = 31	NA	First, second, and third day	The subxiphoid drain better preserves lung function.
					Group 1 (subxiphoid) = 16	56.43 ± 9.04 years		
					Group 2 (intercostal) = 15	59.20 ± 8.4 years		
3	Guden et al. [13]	Akif Ersoy Thoracic & Cardiovascular Surgery Education & Research Hospital, Istanbul, Turkey	December 2010	RCT	Total (N) = 40	NA	Before extubation and one, three, six, 12, 18, 24, and 48 hours after extubation	Subxiphoid and intercostal both have similar clinical outcomes.
					Group 1 (subxiphoid) = 20	57.3 + 11.6 years		
					Group 2 (intercostal) = 20	55.7 + 10.4 years		
4	Cancio et al. [16]	Federal University of São Paulo, São Paulo, São Paulo, Brazil	2012	RCT	Total (N) = 40	NA	First, third, and fifth day	Subxiphoid drain better preserves lung function.
					Group 1 (intercostal) = 19	57.37 ± 10.83 years		
					Group 2 (subxiphoid) = 21	53.86 ± 10.30 years		
5	Guizilini et al. [17]	Federal University of São Paulo, São Paulo, São Paulo, Brazil	2014	RCT	Total (N) = 68	NA	First and fifth day	Subxiphoid drain better preserves lung function.
					Group 1 (intercostal) = 33	57.0 ± 7.7 years		
					Group 2 (subxiphoid) = 35	59.6 ± 8.4 years		
6	Simon et al. [14]	Fundação Universitária de Cardiologia, Porto Alegre, Rio Grande do Sul, Brazil	July 2014 to August 2015	RCT	Total (N) = 48	NA	First, third, and before discharge	There was no difference in maximal respiratory pressures.
					Group 1 (intercostal) = 24	65.58 ± 9.50 years		
					Group 2 (subxiphoid) = 2 4	61.75 ± 9.58 years		
7	Guizilini et al. [23]	São Paulo Hospital, Escola Paulista de Medicina, Federal University of São Paulo, São Paulo, São Paulo, Brazil.	December 2007 and March 2012	RCT	Total (N) = 56	NA	First, third, and fifth postoperative day	Subxiphoid pleural drain has better preservation and recovery of pulmonary capacity and volume.
					Group 1 (intercostal) = 27	59.22 ± 11.73 years		
					Group 2 (subxiphoid) = 29	56.66 ± 10.33 years		
8	Guizilini et al. [11]	Federal University of São Paulo	2004	Retrospective cohort study	Total (N) = 28	57.49 ± 8.40 years	First, third, and fifth day	The subxiphoid technique of drain insertion has demonstrated better preservation of lung function compared to the intercostal drain site.
					Group 1 (intercostal) = 15	57.53 ± 10.29 years		
					Group 2 (subxiphoid) = 13	57.46 ± 5.94 years		
9	Elnasr et al. [8]	Cardiothoracic Surgery Department, Tanta University, Egypt	2017	Retrospective cohort study	Total (N) = 71	NA	Preoperative, one-hour post-extubation, on the third post-extubation day, and predischarge	Subxiphoid and intercostal both have similar clinical outcomes. Analgesia is a requirement for patients with intercostal pleural drainage.
					Group 1 (intercostal) = 38	55.9 ± 8.6 years		
					Group 2 (subxiphoid) = 33	54 ± 7.9 years		

Results of Individual Studies

The outcomes reported in all studies are summarized in Table 2.

**Table 2:** Summary of outcomes CABG, coronary artery bypass grafting; COPD, chronic obstructive pulmonary disease; ECC, extracorporeal circulation; FEV, forced expiratory volume; FVC, forced vital capacity; MEP, maximum expiratory pressure; MIP, maximum inspiratory pressure; OPCAB, off-pump coronary artery bypass grafting; POD, postoperative day; PRE-OP, preoperative; VC, vital capacity

Serial number	Studies	Patients (N)	Time of intubation/mechanical ventilation (hours)		PRE-OP	POD 1	POD 2	POD 3	POD 5	Outcome
1	Hagl et al. [12]	Group 1 (subxiphoid) = 15	12.5 + 4.6 hours	VC (L/MIN)	3.7 ± 0.7 (L/MIN)	1.9 ± 1.0 (L/MIN)	NA	1.8 ± 0.6 (L/MIN)	2.2 ± 0.5 (L/MIN)	Inserting the suxiphoid pleural drain results in significantly less impairment of pulmonary function and subjective pain compared to intercostal insertion.
				% predicted VC	92.3 ± 30.6 (% PRED VC)	45.3 ± 15.00 (% PRED VC)	NA	44.5 ± 15.1 (% PRED VC)	56.9 ± 12.6 (% PRED VC)	
				FEV (L/MIN)	2.8 ± 0.7 (L/MIN)	1.2 ± 0.4 (L/MIN)	NA	1.4 ± 0.4 (L/MIN)	1.6 ± 0.5 (L/MIN)	
				% predicted FEV	86.2 ± 18.2 (% PRED FEV)	36.9 ± 12.0 (% PRED FEV)	NA	42.0 ± 12.1 (% PRED FEV)	50.8 ± 12.1 (% PRED FEV)	
				PO2 (mmHg)	84.4 ± 8.8 (mmHg)	85.2 ± 15.2 (mmHg)	NA	89.4 ± 10.5 (mmHg)	79.7 ± 9.1 (mmHg)	
				PCO2 (mmHg)	39.3 ± 7.4 (mmHg)	42.2 ± 6.5 (mmHg)	NA	39.0 ± 2.4 (mmHg)	41.6 ± 5.4 (mmHg)	
				Pain score (0-10)	NA	NA	NA	Pain score at rest = 1.1 + 0.6	Pain score at rest = 0.8 + 0.6	
								Pain score on forced inspiration = 1.5 + 0.8	Pain score on forced inspiration = 1.1 + 0.5	
		Group 2 (intercostal) = 15	14.0 ± 5.7 hours	VC (L/MIN)	3.6 ± 0.9 (L/MIN)	1.2 ± 0.4 (L/MIN)	NA	1.7 ± 0.7 (L/MIN)	2.3 ± 0.6 (L/MIN)	
				% predicted VC	88.0 ± 18.2 (% PRED VC)	28.6 ± 8.7 (% PRED VC)	NA	42.2 ± 17.6 (% PRED VC)	55.5 ± 14.8 (% PRED VC)	
				FEV (L/MIN)	3.3 ± 1.0 (L/MIN)	1.2 ± 0.7 (L/MIN)	NA	1.8 ± 1.0 (L/MIN)	2.5 ± 1.6 (L/MIN)	
				% predicted FEV	83.5 ± 16.4 (% PRED FEV)	28.0 ± 10.6 (% PRED FEV)	NA	40.6 ± 14.5 (% PRED FEV)	53.9 ± 12.5 (% PRED FEV)	
				PO2 (mmHg)	81.9 ± 9.1 (mmHg)	79.3 ± 9.9 (mmHg)	NA	88.1 ± 7.8 (mmHg)	84.1 ± 7.4 (mmHg)	
				PCO2 (mmHg)	42.3 ± 11.2 (mmHg)	39.0 ± 6.5 (mmHg)	NA	41.1 ± 5.6 (mmHg)	38.3 ± 6.0 (mmHg)	
				Pain score (0-10)	NA	NA	NA	Pain score at rest = 1.2 + 0.8	Pain score at rest = 0.5 + 0.5	
								Pain score on forced inspiration = 1.3 + 0.7	Pain score on forced inspiration = 0.8 + 0.4	
2	Ozelami Vieira et al. [15]	Group 1 (subxiphoid) = 16		VC (L/MIN)	3.25 ± 0.65 (L/MIN)	2.26 ± 0.58 (L/MIN)	NA	2.00 ± 0.55 (L/MIN)	NA	Inserting the drain in the subxiphoid region causes minimal change in lung function and discomfort, leading to improved recovery of respiratory parameters.
				% predicted VC	86.58 ± 7.51 (% PRED VC)	55.51 ± 11.26 (% PRED VC)	NA	47.93 ± 11.93 (% PRED VC)	NA	
				FEV (L/MIN)	2.60 ± 0.49 (L/MIN)	1.73 ± 0.44 (L/MIN)	NA	1.56 ± 0.41 (L/MIN)	NA	
				% predicted FEV	87.50 ± 7.45 (% PRED FEV)	53.61 ± 11.88 (% PRED FEV)	NA	47.57 ± 11.13 (% PRED FEV)	NA	
				PO2 (mmHg)	82.93 ± 9.89 (mmHg)	94.04 ± 17.32 (mmHg)	96.20 ± 15.04 (mmHg)	NA	NA	
				Pain score (0-10)	NA	Pain score at rest = 6.37 ± 1.14	Pain score at rest = 5.62 ± 1.02	Pain score at rest = 4.62 ± 0.95	NA	
						Pain score on forced expiratory effort = 7.68 ± 1.19	Pain score on forced expiratory effort = 6.68 ± 0.87	Pain score on forced expiratory effort = 5.37 ± 1.08		
		Group 2 (intercostal) = 15		VC (L/MIN)	3.12 ± 0.76 (L/MIN)	2.32 ± 0.70 (L/MIN)	NA	2.04 ± 0.56 (L/MIN)	NA	
				% predicted VC	88.23 ± 11.88 (% PRED VC)	62.88 ± 16.70 (% PRED VC)	NA	54.68 ± 14.53 (% PRED VC)	NA	
				FEV (L/MIN)	2.54 ± 0.61 (L/MIN)	1.82 ± 0.53 (L/MIN)	NA	1.64 ± 0.48 (L/MIN)	NA	
				% predicted VC	91.19 ± 11.91 (% PRED VC)	63.09 ± 14.69 (% PRED VC)	NA	56.99 ± 13.92 (% PRED VC)	NA	
				PO2 (mmHg)	81.93 ± 10.65 (mmHg)	97.86 ± 27.50 (mmHg)	NA	NA	NA	
				Pain score (1-10)	NA	Pain score at rest = 8.33 ± 1.17	Pain score at rest = 7.60 ± 1.05	Pain score at rest = 6.20 ± 0.94	NA	
						Pain score on forced expiratory effort = 9.06 ± 0.88	Pain score on forced expiratory effort = 8.86 ± 1.12	Pain score on forced expiratory effort = 7.00 ± 1.13		
3	Guden et al. [13]	Group 1 (subxiphoid) = 20	8.8 ± 4.5 hours	PO2, mmHg	NA	111 (5.76) (mmHg)	105 (4.41) (mmHg)	NA	NA	Both the subxiphoid and intercostal approaches for chest tube placement resulted in comparable clinical outcomes.
				PCO2, mmHg	NA	38 (0.59) (mmHg)	39 (0.69) (mmHg)	NA	NA	
				O2 saturation (%)	NA	96 (0.37) %	97 (0.49) %	NA	NA	
		Group 2 (intercostal) = 20	8.1 ± 4.8 hours	PO2, (mmHg)	NA	111 (5.3) (mmHg)	106 (4.87) (mmHg)	NA	NA	
				PCO2, (mmHg)	NA	37 (0.5) (mmHg)	38 (0.65) (mmHg)	NA	NA	
				O2 saturation	NA	94 (0.49) %	97 (0.52) %	NA	NA	
4	Cancio et al. [16]	Group 1 (intercostal) = 19	10.93 ± 1.25 hours	MIP (CM H2O)	76.89 ± 21.15 (CM H2O)	39.58 ± 12.92 (CM H2O)	NA	44.62 ± 10.89 (CM H2O)	53.09 ± 9.84* (CM H2O)	Patients who underwent subxiphoid pleural drainage demonstrated less decrease in respiratory muscle strength, better preservation of blood oxygenation, and reduced thoracic pain compared to those with intercostal drains in early postoperative OPCAB cases.
				MEP (CM H2O)	93.89 ± 24.70 (CM H2O)	49.89 ± 11.02 (CM H2O)	NA	59.57 ± 9.08 (CM H2O)	66.88 ± 10.90 (CM H2O)	
				PO2 (mmHg)	81.33 ± 9.80 (mmHg)	72.40 ± 11.01 (mmHg)	NA	NA	NA	
				pCO2 (mmHg)	37.40 ± 3.35 (mmHg)	47.73 ± 8.68 (mmHg)	NA	NA	NA	
				Pain score (0-10)		Pain score = 8.73 ± 1.09	NA	Pain score = 7.15 ± 1.06	Pain score = 3.89 ± 1.19	
		Group 2 (subxiphoid) = 21	9.39 ± 1.96 hours	MIP (CM H2O)	81.25 ± 26.49 (CM H2O)	49.96 ± 12.05 (CM H2O)	NA	56.68 ± 16.07 (CM H2O)	67.29 ± 15.62 (CM H2O)	
				MEP (CM H2O)	93.00 ± 30.00 (CM H2O)	61.49 ± 12.07 (CM H2O)	NA	69.59 ± 12.01 (CM H2O)	78.23 ± 12.98 (CM H2O)	
				PO2 (mmHg)	77.67 ± 8.19 (mmHg)	86.21 ± 7.67 (mmHg)	NA	NA	NA	
				pCO2 (mmHg)	38.00 ± 2.94 (mmHg)	39.77 ± 4.02 (mmHg)	NA	NA	NA	
				Pain score (0-10)	NA	Pain score = 6.14 ± 1.49	NA	Pain score = 4.81 ± 1.80	Pain score = 1.95 ± 0.97	
5	Guizilini et al. [17]	Group 1 (intercostal) = 33	11.2 ± 1.9 hours	FVC (L/MIN)	3.3 ± 0.4 (L/MIN)	NA	NA	NA	NA	Subxiphoid pleural drainage resulted in better functional capacity and exercise tolerance, along with a smaller pulmonary shunt fraction and improved clinical outcomes compared to intercostal pleural drainage following off-pump CABG.
				% predicted FVC	95.5 + 12.7 (% PRED FVC)	38.7 ± 9.8 (of preop) (% PRED FVC)	NA	NA	61.9 ± 7.2 (% PRED FVC)	
				FEV (L/MIN)	2.9 ± 0.3 (L/MIN)	NA	NA	NA	NA	
				% predicted (FEV)	101.1 + 11.8 (% PRED FEV)	40.7 ± 8.4 (% PRED FEV)	NA	NA	62.3 ± 5.8 (% PRED FEV)	
				Pain score (0-10)	NA	Pain score = 8.6 ± 1.0	NA	NA	Pain score = 3.7 ± 1.2	
		Group 2 (subxiphoid) = 35	8.8 ± 1.3 hours	FVC (L/MIN)	3.5 ± 0.2 (L/MIN)	NA	NA	NA	NA	
				% predicted FVC	91.5 + 9.4 (% PRED FVC)	50.3 ± 10.3 (% PRED FVC)	NA	NA	75.8 ± 8.6 (% PRED FVC)	
				FEV (L/MIN)	2.9 ± 0.2 (L/MIN)	NA	NA	NA	NA	
				% predicted FEV	97.9 + 7.8 (% PRED FEV)	48.5 ± 7.1 (% PRED FEV)	NA	NA	77.3 ± 8.4 (% PRED FEV)	
				Pain score (0-10)	NA	Pain score = 6.3 ± 1.4	NA	NA	Pain score = 1.8 ± 0.8	
6	Simon et al. [14]	Group 1 (intercostal) = 24	24 (20 ± 27) hours	MIP (CM H2O)	53.83 ± 22.71 (CM H2O)	NA	NA	NA	NA	There was no variation in maximal respiratory pressures concerning the insertion site of the pleural drain among patients who underwent CABG surgery with the use of ECC.
				% predicted MIP	58.93 + 24.91 (% PRED MIP)	NA	NA	NA	NA	
				MEP (CM H2O)	67.50 ± 25.02 (CM H2O)	NA	NA	NA	NA	
				% predicted MEP	38.69 + 14.13 (% PRED MEP)	NA	NA	NA	NA	
				Pain score (0-10)	NA	Pain score = 2.96	NA	Pain score = 1.39	NA	
		Group 2 (subxiphoid) = 24	24.5 (15 ± 44) hours	MIP (CM H2O)	61.46 ± 24.58 (CM H2O)	NA	NA	NA	NA	
				% predicted MIP	63.37 + 20.96 (% PRED MIP)	NA	NA	NA	NA	
				MEP (CM H2O)	73.75 ± 22.95 (CM H2O)	NA	NA	NA	NA	
				% predicted MEP	40.86 + 10.95 (% PRED MEP)	NA	NA	NA	NA	
				Pain score (0-10)		Pain score = 3.54	NA	Pain score = 1.89	NA	
7	Guizilini et al. [23]	Group 1 (intercostal) =27	13.98 ± 1.4 hours	FVC (L/MIN)	3.27 ± 0.26 (L/MIN)	NA	NA	NA	NA	In severe COPD patients, subxiphoid pleural drainage resulted in better preservation and recovery of pulmonary capacity and volumes, with a lower pulmonary shunt fraction and improved clinical outcomes in early postoperative OPCAB.
				% predicted FVC	98.2 ± 21.70 (% PRED FVC)	NA	NA	NA	NA	
				FEV1 (L/MIN)	1.40 ± 0.35 (L/MIN)	NA	NA	NA	NA	
				% predicted FEV1	40.32 ± 7.22 (% PRED FEV)	NA	NA	NA	NA	
		Group 2 (subxiphoid) = 29	16.25 ± 2.1 hours	FVC (L/MIN)	3.51 ± 0.45 (L/MIN)	NA	NA	NA	NA	
				% predicted FVC	101.25 ± 25.24 (% PRED FVC)	NA	NA	NA	NA	
				FEV1 (L/MIN)	1.37 ± 0.22 (L/MIN)	NA	NA	NA	NA	
				% predicted FEV	38.21 ± 9.07 (% PRED FEV)	NA	NA	NA	NA	
8	Guizilini et al. [11]	Group 1 (intercostal) = 15	10.47 ± 1.25 hours	VC (L/MIN)	3.69 ± 0.68 (L/MIN)	NA	NA	NA	NA	The subxiphoid technique of drain insertion has demonstrated better preservation of lung function compared to the intercostal drain site.
				% predicted VC	102.20 ± 16.64 (% PRED VC)	33.36 ± 8.34 (% PRED VC)	NA	45.42 ± 7.06 (% PRED VC)	55.13 ± 8.30 (% PRED VC)	
				FEV (L/MIN)	2.86 ± 0.47 (L/MIN)	NA	NA	NA	NA	
				% predicted FEV	98.89 ± 14.40 (% PRED FEV)	35.70 ± 8.66 (% PRED FEV)	NA	48.04 ± 7.22 (% PRED FEV)	58.80 ± 8.51% (% PRED FEV)	
				PO2 (mmHg)	74.40 ± 6.85 (mmHg)	NA	NA	NA	NA	
				PCO2 (mmHg)	38.27 ± 3.65 (mmHg)	NA	NA	NA	NA	
		Group 2 (subxiphoid) = 13	10.00 ± 0.91 hours	VC (L/MIN)	3.46 ± 1.16 (L/MIN)	NA	NA	NA	NA	
				% predicted VC	96.05 ± 13.72 (% PRED VC)	50.09 ± 14.35% (% PRED VC)	NA	67.00 ± 15.88 (% PRED VC)	78.22 ± 11.90% (% PRED VC)	
				FEV (L/MIN)	2.90 ± 0.97 (L/MIN)	NA	NA	NA	NA	
				% predicted FEV	99.16 ± 15.81 (% PRED FEV)	50.44 ± 13.18 (% PRED FEV)	NA	67.09 ± 15.12% (% PRED FEV)	79.56 ± 11.52% (% PRED FEV)	
				PO2 (mmHg)	77.00 ± 6.75 (mmHg)	NA	NA	NA	NA	
				PCO2 (mmHg)	37.62 ± 4.03 (mmHg)	NA	NA	NA	NA	
9	Elnasr et al. [8]	Group 1 (intercostal) = 38	7 (6 ± 20) hours	PO2/FiO2	388.2 ± 38.9	NA	NA	299.95 ± 92.7	NA	Both post-CABG approaches for pleural drains are effective without compromising pulmonary functions. However, patients with intercostal pleural drains require more analgesia.
		Group 2 (subxiphoid) = 33	9 (6 ± 20) hours	PO2/FiO2	374.9 ± 38.6	NA	NA	334.8 ± 307.4	NA	

Methodological Quality Assessment

The Modified Downs and Black scores were assigned to each study by two authors, and the results are presented in Table 3. Scores ranged from 11 to 18, with the study conducted by Guden et al. [13] receiving the highest score, while the study by Guizilini et al. [11] received the lowest score. Notably, there is a very high and positive correlation (r = 0.92) between the ratings provided by rater 1 and rater 2.

**Table 3:** Modified Down and Black Scale scores and interobserver agreement (Kappa statistics) for the included studies

Study	Rater 1	Rater 2	Kappa
Hagl et al. [12]	16	17	0.92
Ozelami Vieira et al. [15]	18	18	NA
Guden et al. [13]	18	17	NA
Cancio et al. [16]	16	16	NA
Guizilini et al. [17]	16	17	NA
Simon et al. [14]	17	17	NA
Guizilini et al. [23]	16	17	NA
Elnasr et al. [8]	11	12	NA
Guizilini et al. [11]	12	13	NA

Meta-analysis of outcomes

Ventilation Time

**Figure 2:**
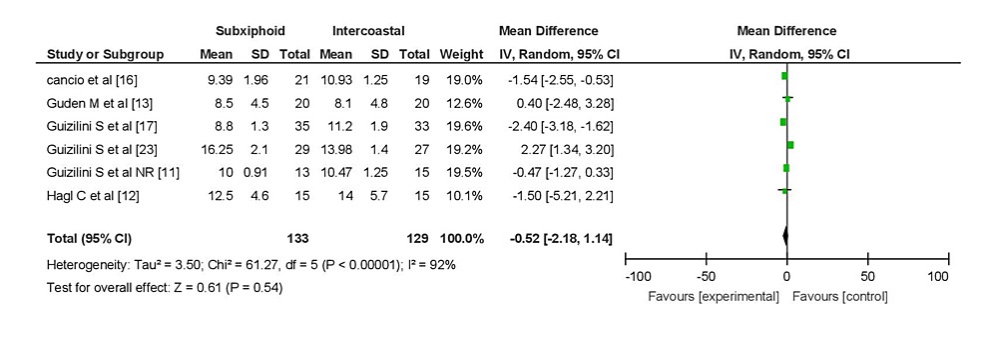
Forest plot for ventilation time

Postoperative ventilation time was assessed in six studies, reported in mean and standard deviation format. A total of 129 patients were included in the intercostal group, while 133 were in the subxiphoid group. The pooled analysis indicated an MD of -0.66 (95% CI (-0.23, -1.08)), signifying shorter ventilation time in the subxiphoid group. However, significant heterogeneity (I2 = 92%) was observed among the studies (Figure 2).

**Figure 3:**
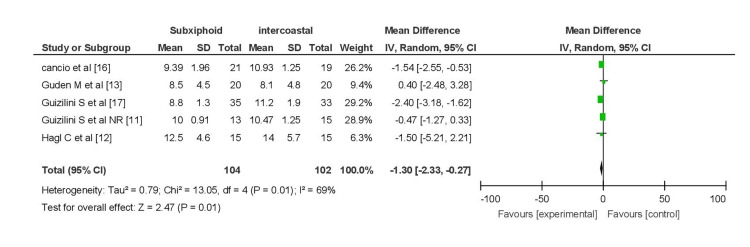
Forest plot for ventilation time excluding patients with COPD COPD, chronic obstructive pulmonary disease

Upon subgroup analysis, excluding the study by Guizilini et al. [23], which exclusively involved chronic obstructive pulmonary disease (COPD) patients undergoing CABG, five studies remained for analysis. In this subset, the subxiphoid group (n = 104) exhibited shorter ventilation time compared to the intercostal group (n = 102), with an MD of -1.43 (95% CI (-1.91, -0.95)). Nonetheless, there was still significant heterogeneity (I2 = 69%) among these studies, but the difference was statistically significant (P = 0.001) (Figure 3).

Pooled analysis was performed after excluding Elnasr et al. [8], Simon et al. [14], and Ozelami Vieira et al. [15], as they did not report ventilation time in the required format.

Pain Score 

**Figure 4:**
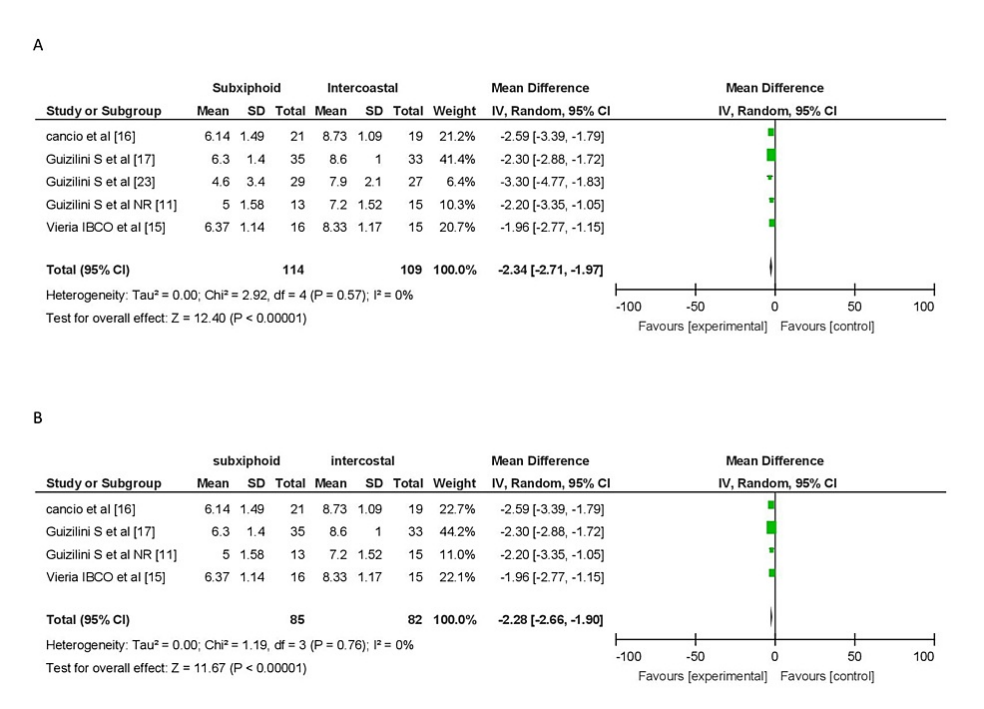
Forest plot for pain score on POD 1 (A) Pain score on POD 1. (B) Pain score on POD 1 excluding patients with COPD. COPD, chronic obstructive pulmonary disease; POD, postoperative day

A pooled analysis of pain scores on postoperative day 1, involving five studies (144 patients in the subxiphoid group and 109 in the intercostal group), revealed a significant difference (MD = -2.34, 95% CI (-2.71, -1.97)), indicating lower pain scores in the subxiphoid group. No heterogeneity was observed (I2 = 0%). Subgroup analysis, excluding the study by Guizilini et al. [23], produced similar results (MD = -2.28, 95% CI (-2.66, -1.90)) with no heterogeneity (I2 = 0%) (Figure 4).

Elnasr et al. [8], Simon et al. [14], Hagl et al. [12], and Guden et al. [13] were not included in the analysis due to reasons such as missing pain score data or different findings. Notably, Elnasr et al. [8] reported higher tramadol use in the intercostal group, while Simon et al. [14] found no significant difference in subjective pain between the two groups. Hagl et al. [12] concluded that the subxiphoid group experienced significantly less pain during forced inspiration on postoperative day one.

**Figure 5:**
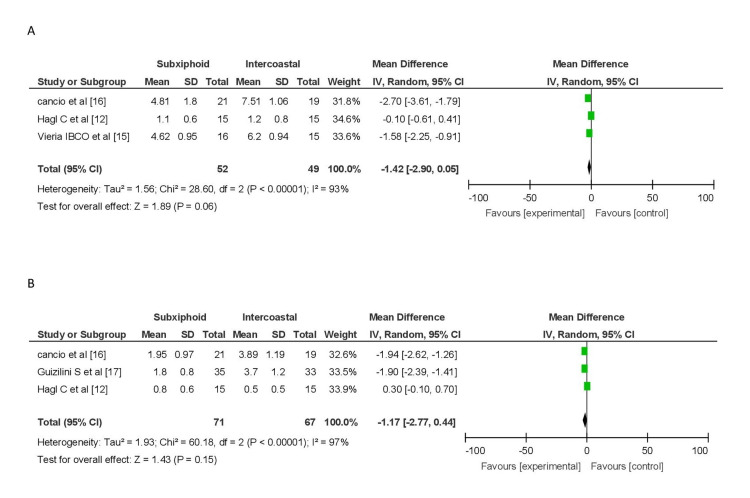
Forest plot for pain score on POD 3 and POD 5 (A) Forest plot of pain score on POD 3. (B) Forest plot of pain score on POD 5. POD, postoperative day

Pooled analysis of pain scores on postoperative day 3, involving three studies (52 patients in the subxiphoid group and 49 in the intercostal group), indicated lower pain scores in the subxiphoid group (MD = -0.98, 95% CI (-1.35, -0.16)). However, significant heterogeneity was observed (I2 = 93%). Similarly, on postoperative day 5, pooled analysis from three studies (71 patients in the subxiphoid group and 67 in the intercostal group) showed lower pain scores in the subxiphoid group (MD = -0.81, 95% CI (-1.09, -0.53)), but with significant heterogeneity (I2 = 97%). These results should be interpreted cautiously due to their heterogeneity (Figure 5).

Guden et al. [13] evaluated the effects of analgesia using Visual Analogue Scale and Verbal Rating Scale scores and found no significant differences in the cumulative doses of necessary rescue analgesics between the groups. The low pain scores in both groups suggested reduced pulmonary morbidity when effective analgesia was provided.

PO2

**Figure 6:**
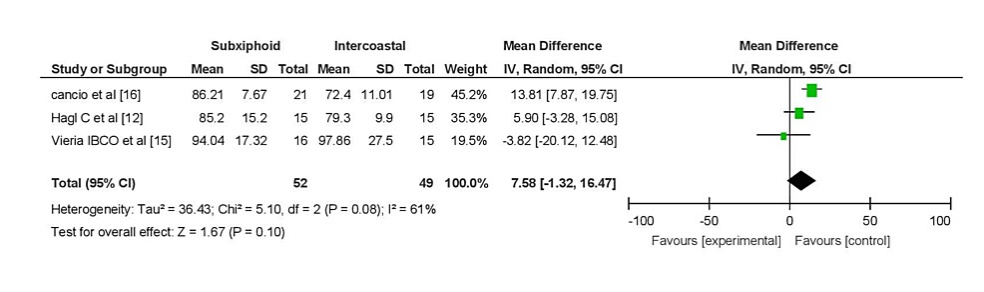
Forest plot for PO2 on POD 1 PO2, partial pressure of oxygen; POD, postoperative day

On postoperative day 1, pooled analysis of three studies (Hagl et al. [12], Ozelami Vieira et al. [15], and Cancio et al. [16]) measuring PO2 in ABG (52 patients in the subxiphoid group and 49 in the intercostal group) suggested a greater decrease in PO2 in the intercostal group (MD = 10.17, 95% CI (5.40, 14.94)), with moderately significant heterogeneity (I2 = 61%) (Figure 6).

Percentage Predicted VC and FEV

A pooled analysis of studies by Guizilini et al. [11,23], Hagl et al. [12], and Ozelami Vieira et al. [15] comparing the decrease in % predicted VC, involving 79 patients in the subxiphoid group and 78 in the intercostal group, revealed a greater decrease in VC in the intercostal group (MD = 10.91, 95% CI (7.36, 14.46)). However, there was significant heterogeneity among the studies (I2 = 81%).

**Figure 7:**
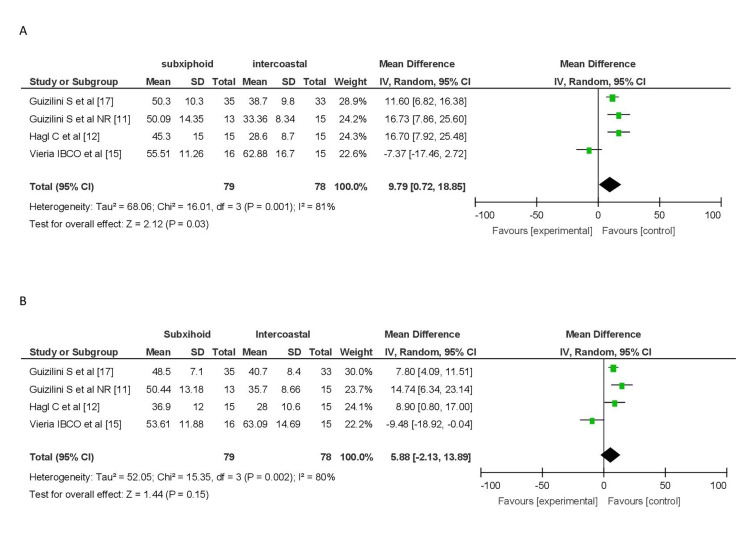
Forest plot for the decrease in percentage predicted VC and FVC (A) Forest plot for the decrease in percentage predicted VC. (B) Forest plot for decrease in percentage predicted FVC. FVC, forced vital capacity; VC, vital capacity

Similarly, pooled analysis of the decrease in % predicted FEV from the same four studies, involving 79 patients in the subxiphoid group and 78 in the intercostal group, showed a greater decrease in predicted FEV in the intercostal group (MD = 7.11, 95% CI (4.14, 14.14)), with significant heterogeneity (I2 = 80%) (Figure 7).

Discussion

Numerous factors can compromise pulmonary function following CABG surgery involving the left internal mammary artery (LIMA). Some of these studies have examined technical questions regarding chest tube placement and its impact on postoperative pulmonary function. This meta-analysis represents the most comprehensive comparison to date of the clinical outcomes associated with subxiphoid and intercostal pleural drain insertion in CABG patients. Nine studies, including seven randomized and two nonrandomized trials, met the inclusion criteria, encompassing a total of 412 patients. Subgroup analysis of the included studies revealed several favorable outcomes in the subxiphoid group, including reduced pain scores, shorter ventilation time, improved spirometry parameters (VC and FEV), and a higher partial pressure of O2 in postoperative ABG analysis.

Pulmonary function is profoundly affected following cardiac surgery. General anesthesia during the procedure results in diminished respiratory muscle tone, decreased lung compliance, and increased total respiratory system resistance [24]. Median sternotomy further compounds these issues by impairing lung function, causing pain, and altering chest wall compliance. This surgical approach can delay lung function recovery and increase morbidity during the early postoperative period [25]. The use of the LIMA can exacerbate pulmonary dysfunction due to the more frequent opening of the pleura and the consequent need for pleural drainage [26,27]. Additionally, the removal of the internal mammary artery can reduce blood supply to the phrenic nerve, further contributing to postoperative pulmonary dysfunction [28]. All these factors contribute to a higher incidence of atelectasis, rendering patients more susceptible to hypoxic pulmonary complications, primarily pneumonia [12]. Sensoz et al. [29] demonstrated that intercostal pleural drains and subxiphoid drains had similar drainage capacity for pleural effusion but that subxiphoid drains reduced the incidence of atelectasis during the CABG postoperative period.

Chest tubes inserted and exteriorized in the intercostal space come into contact with the highly sensitive periosteum and parietal pleura. This contact can lead to ventilator-dependent pain, limiting deep inspiration and forcing patients to adopt antalgic postures, resulting in immobilization and reduced lung volumes and capacities [30]. Intercostal placement of the drain can irritate the intercostal nerves due to the large bore drain tube, increasing pain [12]. In contrast, subxiphoid drain placement does not breach the intercostal space, resulting in less pain.

This meta-analysis suggests that the preservation of respiratory muscle strength and oxygenation appears to be partly responsible for the shorter intubation time and reduced pain observed in patients with subxiphoid drains compared to intercostal drains. Moreover, the lesser interference with respiratory mechanics by subxiphoid drains leads to better preservation of spirometry parameters, enhancing patient compliance with chest physiotherapy and promoting earlier functional recovery of the lungs.

Limitations

There are some limitations to this study. Notably, Simon et al. [14] conducted elective CABG with extracorporeal circulation, while the other studies included patients who underwent off-pump CABG. The study by Guizilini et al. [23] included patients exclusively with coronary artery disease and COPD, whereas such patients were not included in the other studies. Additionally, the assessment of pulmonary function was not uniform across all studies, with some measuring VC and FEV while Simon et al. [14] and Cancio et al. [16] measured maximal inspiratory pressure and maximal expiratory pressure.

## Conclusions

This meta-analysis provides evidence that patients who undergo CABG with left pleural subxiphoid drain placement experience shorter ventilation times, lower pain scores, better postoperative percentages of predicted VC and FVC, and a higher partial pressure of O2 in ABG compared to those with intercostal drain placement. These findings support the notion that subxiphoid left pleural drainage after CABG is associated with less discomfort and better respiratory outcomes when compared to intercostal drainage.
